# Comparative Prognostic Role of PLR and NLR in Colon Cancer: A Retrospective Analysis of Preoperative Inflammatory Markers

**DOI:** 10.3390/medicina61091580

**Published:** 2025-08-31

**Authors:** Roxana Loriana Negrut, Adrian Cote, Bogdan Feder, Florian Dorel Bodog, Adrian Marius Maghiar

**Affiliations:** Department of Surgical Disciplines, Faculty of Medicine and Pharmacy, University of Oradea, 410073 Oradea, Romania; popa.roxanaloriana@student.uoradea.ro (R.L.N.); bogdanfeder@yahoo.com (B.F.); fbodog@gmail.com (F.D.B.); amaghiar@uoradea.ro (A.M.M.)

**Keywords:** colon cancer, inflammatory markers, platelet–lymphocyte ratio, PLR, neutrophil–lymphocyte ratio, NLR, tumor, prognostic marker

## Abstract

Systemic inflammation plays a key role in cancer progression, and markers such as neutrophil-to-lymphocyte ratio (NLR) and platelet-to-lymphocyte ratio (PLR) have gained attention as potential prognostic tools in colorectal cancer. However, their comparative utility in colon cancer remains unclear. *Objective*: This study was aimed to assess and compare the prognostic value of preoperative NLR and PLR in evaluating tumor aggressiveness in colon cancer patients undergoing elective surgery. *Methods*: We conducted a retrospective observational study on 64 patients with histologically confirmed colon cancer treated between 2019 and 2022. Only elective cases were included; rectal and emergency surgeries were excluded. Demographic, clinical, pathological, and laboratory data were collected. Tumor aggressiveness was assessed based on tumor size, histologic grade, lymphovascular and perineural invasion, and lymph node involvement. Statistical analysis included Pearson correlation, ANCOVA, logistic regression, and principal component analysis (PCA). *Results*: PLR showed a significant positive correlation with tumor size (r = 0.428, *p* < 0.001) and tumor stage (r = 0.314, *p* = 0.012), whereas NLR did not. Logistic regression and PCA indicated that PLR better reflected tumor burden, while NLR was more associated with systemic inflammation. Neither marker significantly predicted postoperative complications or in-hospital mortality. *Conclusions*: PLR may serve as a useful, non-invasive biomarker for assessing tumor aggressiveness in colon cancer, supporting its integration into preoperative risk stratification. The results from this single-center, retrospective cohort showed moderate associations between PLR and tumor size and stage, whereas NLR did not. These findings are hypothesis-generating and insufficient for clinical implementation; prospective, adequately powered studies with survival endpoints are required.

## 1. Introduction

In today’s world, colon cancer is still one of the most prevalent malignancies, remaining one of the leading causes of cancer-related deaths.

Despite significant advancements in medical technologies, the 5-year survival rates after surgical and oncological treatment remain suboptimal. According to the National Institutes of Health, the 5-year survival rate for stage I colorectal cancer is 91.1%, rapidly decreasing as the disease becomes more advanced to 15.7% for colorectal cancer with metastasis [1].

Systemic inflammatory markers such as the neutrophil-to-lymphocyte ratio (NLR) and platelet-to-lymphocyte ratio (PLR) have emerged as accessible, low-cost tools with potential prognostic significance in various malignancies, including colorectal cancer. These biomarkers are routinely available from standard blood tests, making them attractive candidates for inclusion in preoperative evaluation protocols.

The role of inflammatory biomarkers in colorectal cancer has been extensively studied over the years. Some studies have reported that elevated inflammatory markers are associated with an increased risk of developing colorectal cancer, particularly colon cancer, although they are not considered reliable early detection markers [2]. Conversely, other studies indicate that the systemic inflammatory cell ratios, particularly the neutrophil-to-lymphocyte ratio, undergo dynamic changes as the disease progresses, suggesting their potential utility in early diagnosis [3,4].

While previous studies focused on the inflammatory biomarkers, particularly NLR, as prognostic indicators of colorectal cancer, the comparative prognostic value for NLR and PLR remains unclear. Numerous studies mention that NLR is a simple, easy-to-obtain, and cost-effective parameter that could be used as a significant predictor of survival in colorectal cases. In some studies, this was found to be a superior indicator of prognosis over other inflammatory markers, such as platelet-to-lymphocyte ratio (PLR) and lymphocyte-to-monocyte ratio (LMR) [5,6,7].

Additionally, there are studies that investigated NLR and PLR as potential diagnostic markers for metastasis. A study that included 1163 colorectal patients concluded that the combination of these inflammatory markers with traditional tumor markers improved the diagnostic accuracy for the detection of metastasis [8]. A systematic review published in 2023 focused on the clinical utility of NLR, LMR, and PLR in colorectal cancer [9].

While the individual role of NLR or PLR in colorectal cancer prognosis has been addressed in previous studies, their direct comparative value remains underexplored, particularly within well-defined and homogeneous patient cohorts. Moreover, the confounding impact of rectal tumors and emergency surgeries on systemic inflammation is often overlooked, despite known variations in tumor biology and inflammatory response.

This study aims to assess and compare the prognostic significance of preoperative PLR and NLR in evaluating tumor aggressiveness in colon cancer. By excluding rectal and emergency cases, we focus on a uniform cohort treated with elective surgery, thus minimizing confounding factors. In addition to classical statistical analysis, we applied principal component analysis (PCA) to explore the relationship between systemic inflammation and tumor burden, providing new insight into the clinical interpretation of these markers. This study focuses on the association between inflammatory markers (PLR and NLR) and tumor aggressiveness in colon cancer patients, specifically analyzing their relationship with tumor size, tumor differentiation, perineural and lymphovascular invasion, and regional node involvement. This study does not propose PLR or NLR as a diagnostic marker for colon cancer.

Recent literature highlighted the growing significance of these markers in assessing tumor characteristics and prognosis in colon cancer. Incorporating PLR into routine screening protocols may help identify patients at risk, potentially contributing to a more personalized approach to treatment.

Future multicenter collaborations will be essential to confirm these findings and establish the role of inflammatory markers in standard cancer care. Expanding research to include circulating tumor cells or T-cell markers could further enhance prognostic precision and patient management.

While previous studies have investigated inflammatory markers in cancer prognosis, this study provides a direct comparison between NLR and PLR in colon cancer aggressiveness and applies PCA analysis to separate systemic inflammation from tumoral load, offering novel statistical insights.

To our knowledge, this is the first study to directly compare PLR and NLR in a colon-only cohort, explicitly excluding rectal and emergency cases, and to apply principal component analysis (PCA) to differentiate systemic inflammation from tumor-specific pathological factors.

Given the heterogeneity of published cut-offs and limited outcome-anchored validation, our analysis is exploratory and intended to generate hypotheses for prospective studies.

## 2. Materials and Methods

A retrospective observational study was conducted, using clinical data collected on a cohort of 64 patients diagnosed with colon cancer and treated at one surgical department from our institution (Clinical Emergency Hospital Bihor, Oradea, Romania). Patient records were reviewed for demographic, clinical, and laboratory characteristics, including age, length of hospital stay, tumor size, histopathological characteristics, surgical approaches, and laboratory markers such as neutrophil–lymphocyte ratio (NLR), platelet–lymphocyte ratio (PLR), hemoglobin (HGB), preoperative white blood cell count (WBC), and others. The study was conducted on data available from January 2019 until December 2022. This study aimed to evaluate whether the PLR and NLR, which are easily measurable markers through blood tests, can provide a non-invasive approach for assessing tumor aggressiveness and staging and as secondary outcomes to predict potential postoperative complications, mortality during clinical care, or the length of hospital stay. The blood samples included were collected preoperatively (the day of surgery or one day before surgery) and after an overnight fast.

### 2.1. Data Collection

The data were gathered from patient records, covering both preoperative and postoperative measurements. The key variables included age; sex; tumor characteristics such as tumor size and positive lymph nodes; surgical details, including type of surgery performed, complications, and bowel restoration; laboratory markers such as NLR and PLR; complications; secondary intervention; and outcomes like length of stay and death during hospital stay.

### 2.2. Inclusion and Exclusion Criteria

Patients included in the study were those with a confirmed diagnosis of colon cancer who underwent elective surgical intervention. The following cases were excluded: patients who underwent emergency surgery due to the potential acute impact on inflammatory markers, patients with tumoral recurrence, and patients whose tumors were deemed unresectable. No rectal or rectosigmoid junction tumors were included in this study. Only colon cancer cases were selected to ensure a homogeneous cohort, with consistent surgical and pathological criteria.

### 2.3. Sampling Method

The sampling process was consecutive, including all eligible patients who met the inclusion and exclusion criteria during the specified timeframe. This approach ensured an unbiased and comprehensive patient selection process.

### 2.4. Statistical Analysis

All statistical analyses were conducted using JASP software (version 0.12.1, Apple Silicon, JASP Team, University of Amsterdam, The Netherlands) and Microsoft Excel for Mac (version 16.90.2, Microsoft Corporation, Redmond, WA, USA).

Descriptive statistics were used to continuous variables, which were reported as means and standard deviations, and categorical variables, reported as frequencies and percentages. The descriptive characteristics of the patients, such as age, length of stay, and laboratory values, were summarized using means, standard deviations, and interquartile ranges. Frequencies for categorical variables such as sex, surgical intervention, bowel continuity, and presence of metastases were calculated to illustrate the distribution of the cohort.

Pearson correlation analysis was used to assess relationships between laboratory markers (e.g., NLR and PLR) and clinical outcomes such as tumor size, staging, length of hospital stay, and complications. Significant correlations were set at standard values, *p* < 0.05.

Tumor aggressiveness in this study was operationally defined by a composite of the following pathological features: tumor size, T stage, N stage, histologic grade, perineural invasion, and lymphovascular invasion.

An analysis of covariance (ANCOVA) was performed to evaluate the effects of independent variables such as NLR, PLR, mortality during hospital stay, and surgical complications on the dependent variable (length of hospital stay). Levene’s test was used to confirm the homogeneity of variances (*p* > 0.05). The model included key covariates like age, sex, and data regarding the tumoral extension (perineural invasion and lymphovascular invasion), which were also evaluated for interaction terms where applicable. No adjustments were made for comorbidities that could affect inflammatory markers (such as diabetes and cardiovascular disease). No patient in the cohort had a documented history of active hematologic disorders, autoimmune diseases, or active infections at the time of surgery. Additionally, no patients with such conditions in remission were identified within the study period. None of the patients included in this study received neoadjuvant chemotherapy or radiotherapy. All cases were treated with primary surgical resection, and patients were only referred for oncologic therapy postoperatively based on final histopathological staging and multidisciplinary team (MDT) evaluation. This approach minimized potential bias from systemic treatments known to influence inflammatory biomarkers.

PLR and NLR were analyzed continuously and, for descriptive purposes, using literature-informed exploratory thresholds (e.g., PLR ≈ 150, NLR ≈ 3). These thresholds were hypothesis-generating and not validated within our cohort.

In order to reduce dimensionality and identify the underlying patterns of the data, a principal component analysis (PCA) was conducted. Promax rotation was used to allow for correlated components. The components that presented Eigenvalues greater than 1 were retained. RC1 was primarily characterized by high loadings from tumor grade, tumor size, and the number of infiltrated lymph nodes, suggesting that this component represents tumor burden and aggressiveness. RC2 was strongly associated with the NLR, indicating that this component primarily reflects systemic inflammation, independent of direct tumor-related factors. PCA provides unique insights beyond traditional correlation methods by revealing underlying patterns in the relationship between inflammatory markers and tumoral characteristics. PCA can uncover broader patterns of variability and identify clusters of related variables. This approach allowed for a better identification of key markers that could be used in further predictive modeling.

Sampling adequacy for PCA was borderline (KMO = 0.518), with low MSA for NLR, indicating limited suitability for dimensionality reduction. We therefore pre-specified PCA as exploratory and interpret components descriptively.

Logistic regression models were developed to predict binary outcomes such as the presence of surgical complications and death during the hospital stay. Predictors included NLR, PLR, age, sex, and tumor-related variables such as infiltrated ganglions and staging. Model performance was assessed using Nagelkerke R^2^ and model diagnostics, including multicollinearity (assessed using variance inflation factor [VIF]), which indicated no severe multicollinearity issues.

This methodological framework ensures a robust statistical evaluation of the relationship between inflammatory markers and tumor aggressiveness in colon cancer patients.

Generative artificial intelligence (ChatGPT, OpenAI version 4.0 and 5.0) was used to assist in the refinement of the English used, organization of scientific ideas, abstract formulation, and phrasing improvements throughout the manuscript. No content, data, or results were generated by AI.

## 3. Results

The study included a cohort of 64 patients with histologically confirmed colon cancer. The mean age of the cohort was 70.47 years (SD = 10.41), with an interquartile range (IQR) of 12.5 years, indicating the predominance of elderly patients with a relatively homogeneous age distribution. The youngest patient was 38 years old, while the oldest was 90 years old. The length of hospital stay ranged from 3 to 44 days, with a mean of 11.95 days (SD = 6.09), reflecting variations in the recovery process related to surgical complications and other clinical factors. The majority of the patients were male (65.6%), with 34.4% female. Patients predominantly resided in urban areas (64.1%), while 35.9% were from rural locations.

The mean tumor size was 5.61 cm (SD = 2.70), with a median IQR of 3.625 cm. The smallest tumor measured 1.7 cm, while the largest reached 14 cm. The number of positive lymph nodes varied significantly, ranging from none to a maximum of 12 ganglions, with a mean of 1.46 (SD = 2.60) and an IQR of 2 ganglions. Among the tumors, 64.1% were not infiltrated by cancerous cells, while 35.9% showed lymph node infiltration.

Regarding tumor staging, the majority of patients were diagnosed with stage II (35.9%) or stage III (28.1%) cancer, with 18.8% in stage I and 17.2% in stage IV. This distribution highlights a significant portion of patients with advanced disease, emphasizing the importance of early diagnosis and effective biomarker identification.

Tumor localization was as follows: 45.3% in the sigmoid colon, 17.2% in the ascending colon, 17.2% in the transverse colon, 14.1% in the caecum, and 6.3% in the descending colon. The sigmoid colon was therefore the most frequent tumor site, consistent with typical epidemiological findings in colorectal cancer. Because none of the tumors involved the rectum or rectosigmoid junction, anterior resection was not indicated.

Three types of surgical procedures were performed: segmental resection (50%), right colectomy (39.1%), and left colectomy (10.9%). Intestinal continuity was restored by anastomosis in 81.3% of cases, while 18.8% of patients required a stoma. Among anastomosis procedures, the end-to-end type was the most commonly used (78.8%), followed by side-to-side (15.4%) and end-to-side (5.8%) techniques.

A total of 7.8% of patients presented with surgical complications. Anastomotic fistulas were observed in 4.7% of cases, with one occurrence each in right colectomy, segmental resection, and left colectomy. Evisceration occurred in 3.1% of patients. Overall, 92.2% of patients had no surgical complications, indicating a generally favorable surgical outcome. Four patients (6.3%) died during the hospital stay, highlighting the importance of identifying reliable prognostic markers to mitigate adverse outcomes.

Metastasis was observed in 12.5% of patients as liver involvement, 7.8% as pulmonary metastasis, and 3.1% as peritoneal spread. Additionally, local invasion was present in 17.2% of the patients, which correlates with the advanced disease stage seen in a subset of patients. This reinforces the need to explore systemic inflammatory markers as potential predictors of metastatic spread and local invasiveness. No patient underwent metastasectomy, and no neoadjuvant chemotherapy was administered prior to surgery.

Histopathological examination revealed that 59.4% of cases were classified as G2 (moderate differentiation), 14.1% as G3 (poor differentiation), and 4.7% as G1 (well-differentiated). The histopathological type of “not otherwise specified” (NOS) represented 76.6% of the cases, while mucinous and multifocal tumors represented 21.9% and 1.6%, respectively. Perineural invasion was present in 31.3% of cases, while lymphovascular invasion was noted in 40.6% of patients. The presence of these features is associated with more aggressive tumors, suggesting a potential role for systemic inflammatory markers in reflecting underlying biological aggressiveness. The data are presented in Table 1.

The mean values, standard deviations, and interquartile ranges for key clinical measures are summarized in Table 2. The same data for blood markers are presented in Table 3.

These descriptive statistics provide a foundational understanding of the population characteristics and form the basis for further exploration of associations between NLR, PLR, and clinical outcomes. All blood samples were collected in the preoperative period (1 day before surgery or on the day of surgery) following a minimum fasting period of 8 h.

Pearson correlation analysis was conducted to determine the relationship between NLR, PLR, and tumor characteristics, including tumor size, staging, and grading. NLR demonstrated a significant moderate positive correlation with PLR (r = 0.536, *p* < 0.001), indicating that these markers are interrelated due to their shared association with systemic inflammation. However, no significant correlation was observed between NLR and tumor size (r = 0.079, *p* = 0.537), suggesting that NLR alone may not serve as an effective biomarker for predicting tumor dimensions in colon cancer. Conversely, PLR was found to be significantly associated with tumor size (r = 0.428, *p* < 0.001), indicating that higher PLR levels could reflect larger tumor dimensions. Also, PLR demonstrated a moderate positive correlation with tumor staging (r = 0.314, *p* = 0.012). These findings suggest that PLR could serve as a valuable indicator for preoperative evaluation of tumor progression and aggressiveness. The ability of PLR to correlate with both tumor size and staging underscores its potential as a non-invasive biomarker for evaluating tumors in colon cancer patients, particularly when considering preoperative risk stratification. The correlation between NLR or PLR and the presence of metastasis is not statistically significant in this study. The data can be visualized in Table 4.

To further investigate the role of NLR and PLR, an ANCOVA was performed to assess their impact on the length of hospital stay while adjusting for key covariates, including surgical complications and in-hospital mortality. There was no significant correlation between PLR and length of stay (F = 0.002, *p* = 0.961), and similarly, NLR did not show a significant effect (F = 0.061, *p* = 0.806). Instead, the presence of surgical complications (F = 87.189, *p* < 0.001) and death during hospitalization (F = 17.511, *p* < 0.001) were found to be significant predictors of extended hospitalization. These results suggest that inflammatory markers such as NLR and PLR may have limited utility in predicting immediate postoperative outcomes such as length of stay in the context of colorectal cancer. The ANCOVA analysis results are presented in Table 5 and Table 6.

Logistic regression was used to determine the predictive capacity of NLR and PLR, along with other clinical covariates such as tumor-related factors. The model summary showed that the inclusion of additional variables improved model fit, as indicated by the lower deviance in Model M_1_ compared to that in M_0_, though the overall explanatory power (Nagelkerke R^2^ = 0.129) remained modest.

The results indicated that tumoral infiltrated lymph nodes, length of hospital stay, and in-hospital mortality were not significantly associated with NLR cut-off levels. The complication binary variable was the only predictor that reached statistical significance (Wald statistic = 4.495, *p* = 0.034), suggesting that complications during hospitalization were significantly associated with NLR levels.

The logistic regression model examining perineural invasion did not show a statistically significant relationship with either NLR or PLR, indicating that these markers alone may not be reliable indicators of perineural involvement. Similarly, the logistic regression model assessing lymphovascular invasion did not find significant associations with NLR or PLR. The odds ratios for NLR and PLR were close to 1.0, suggesting a limited effect size in predicting lymphovascular involvement.

A separate logistic regression analysis was performed to evaluate the potential of NLR, PLR, and other clinical markers in predicting mortality during the hospital stay. The results showed that none of the inflammatory markers were statistically significant predictors of death (*p* = 0.845). This suggests that NLR and PLR alone may not be strong prognostic indicators for in-hospital mortality.

A linear regression model was constructed to examine factors associated with the length of hospital stay, incorporating NLR, PLR, tumor staging, age, and sex as covariates. The overall model did not account for a large portion of the variability in hospital stay (adjusted R^2^ < 0.3), and neither NLR nor PLR emerged as a significant predictor. Instead, the primary factors influencing hospital stay were related to postoperative complications and patient demographics.

In order to identify underlying patterns among clinical markers, a principal component analysis (PCA) was performed on a set of inflammatory and tumor-related variables, with Promax rotation applied to allow for correlated components. PCA was used in an exploratory context to reduce variable complexity and to identify potential patterns. RC1 was primarily associated with tumor characteristics, while RC2 appeared to reflect systemic inflammation. The analysis extracted two principal components (RC1 and RC2), together explaining 64.4% of the total variance. RC1, accounting for 37.8% of the variance, was primarily characterized by high loadings from tumor grade (0.866), tumor size (0.495), and tumor-infiltrated lymph nodes (0.660), suggesting that this component represents tumor burden and aggressiveness. RC2, explaining 26.6% of the variance, was strongly associated with the NLR (loading = 1.426), indicating that this component primarily reflects systemic inflammation, independent of tumor-related factors. The low uniqueness value of NLR (0.143) suggests that the extracted components effectively captured its variance. These findings, presented in Table 7, highlight a moderate correlation between inflammatory markers and tumor-related variables, suggesting that while systemic inflammation and tumor aggressiveness are partially linked, they also contribute independently to disease progression and prognosis.

**Figure 1:**
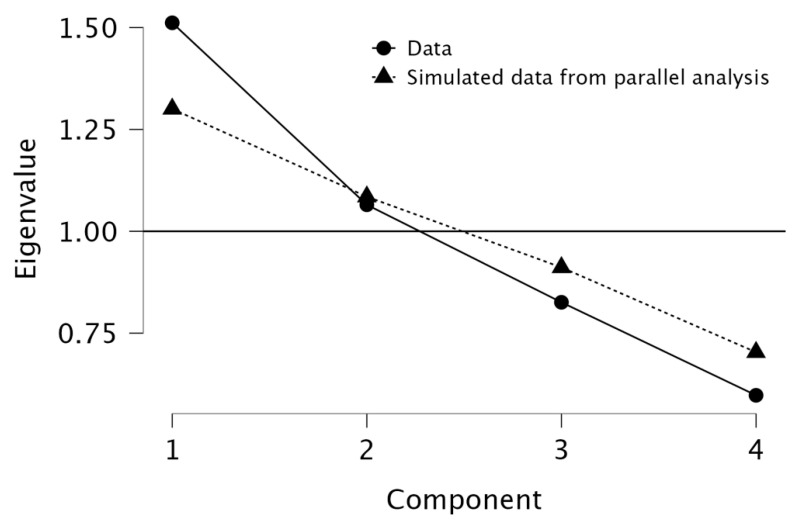
Scree plot for principal component analysis of inflammatory and tumor-related variables.

**Figure 2:**
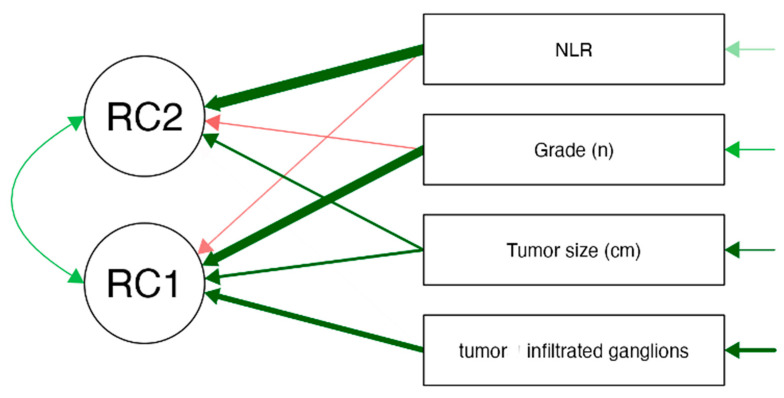
Component loading plot for principal component analysis (RC1, RC2). Green arrows indicate positive loadings, red arrows indicate negative loadings.

The scree plot generated during the PCA analysis was used to determine the appropriate number of components to retain. The plot displayed a noticeable elbow at Component 2, indicating that the majority of the variance in the data could be effectively explained by the first two components. Component 1 had an eigenvalue of 1.512 (37.8% of the variance), while Component 2 had an eigenvalue of 1.065 (26.6% of the variance), together explaining 64.4% of the total variance. The path diagram further supports this finding, with RC1 (Principal Component 1) primarily associated with tumor-related characteristics, including tumor grade (loading = 0.866), tumor size (loading = 0.495), and infiltrated lymph nodes (loading = 0.660). This suggests that RC1 represents tumor burden and aggressiveness. Meanwhile, RC2 (Principal Component 2) was strongly correlated with the NLR (loading = 0.960), indicating that this component predominantly captures systemic inflammation. The final decision to retain both components (RC1 and RC2) was based on their significant contribution to explaining data variability and their distinct biological relevance. The findings emphasize that tumor burden and systemic inflammation are related but independent factors in colon cancer, reinforcing the hypothesis that inflammatory markers like NLR are distinct from direct tumor characteristics but still play a role in disease progression. See Figure 1 and Figure 2 for visualization.

To assess the suitability of principal component analysis (PCA) for dimensionality reduction, the Kaiser–Meyer–Olkin (KMO) test and Bartlett’s test of sphericity were performed. The overall KMO measure was 0.518, indicating borderline adequacy for PCA, with individual MSA values for infiltrated lymph nodes (0.615), tumor size (0.528), grade (0.504), and NLR (0.419). The low KMO value for NLR suggests it may not contribute effectively to the PCA model, potentially influencing the robustness of the extracted components. Bartlett’s test of sphericity was significant (χ^2^ = 14.004, df = 6, *p* = 0.030), confirming that the correlation matrix was appropriate for PCA. Parallel analysis further validated the retention of two principal components (χ^2^ = 15.550, df = 2, *p* < 0.001), supporting the division of tumor burden-related and systemic inflammatory components. Despite the borderline KMO value, Bartlett’s test was significant, indicating that PCA could still be performed. However, the low MSA value for NLR suggests that its contribution to PCA is limited; therefore, the component formation should be interpreted with caution.

Exploratory factor analysis (EFA) was conducted to identify underlying latent constructs among the variables. The analysis extracted two primary factors, which together explained 36.4% of the total variance. Factor 1 (23.3% of the variance) had strong loadings from tumor-related characteristics, including tumor grade (0.874), tumor size (0.730), and the number of infiltrated ganglions (0.885), suggesting that this factor represents tumor aggressiveness. Factor 2 (12.9% of the variance) was primarily associated with NLR (0.669), indicating that it reflects systemic inflammation. Although the extracted factors explain 36.4% of the total variance, the results suggest that additional inflammatory markers, such as C-reactive protein or interleukin-6, might refine predictive models for tumor aggressiveness.

Further, we investigated the relationship between clinical characteristics and the probability of detecting an NLR value under 3, a threshold supported by the existing literature [7,10,11,12]. The first model investigated the relationship between tumor size and the probability of having NLR under 3. The parameter estimates indicated a negative intercept of −0.127 and a negative coefficient for tumor size (β = −0.122), suggesting a significant negative relationship between the tumor size and the likelihood of detecting an NLR value under 3. The detection plot (left panel) shows that as tumor size increases, the probability of NLR being under a value of 3 significantly declines. This suggests that patients with larger tumors exhibit a stronger systemic inflammatory response, potentially indicating a more aggressive disease. For smaller tumors, the probability of maintaining a low NLR remains high, which may be associated with a less pronounced inflammatory state and potentially better prognosis.

**Figure 3:**
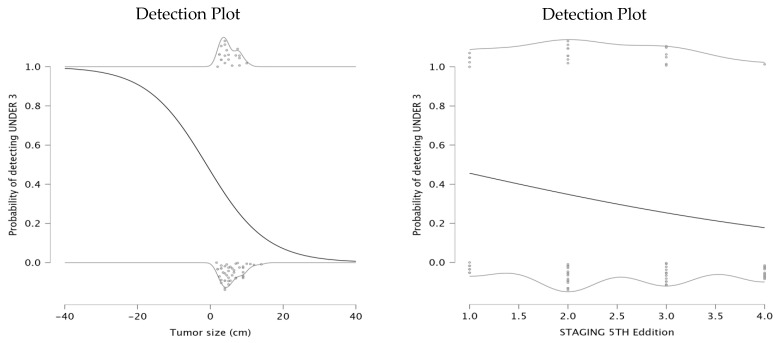
Detection plot, probability based on tumor size and detection probability based on staging.

The second model explored the relationship between staging and the probability of detecting NLR under 3. The parameter estimates showed an intercept of 0.277 and a negative coefficient for staging (β = −0.452), indicating that a higher tumor stage is associated with a decreased probability of NLR remaining below 3. The detection plot (right panel) demonstrates a downward trend in probability as staging increases. This findings aligns with the hypothesis that higher tumor stages are more likely to provoke a systemic inflammatory response, leading to elevated NLR levels. The detection plots are presented in Figure 3.

**Figure 4:**
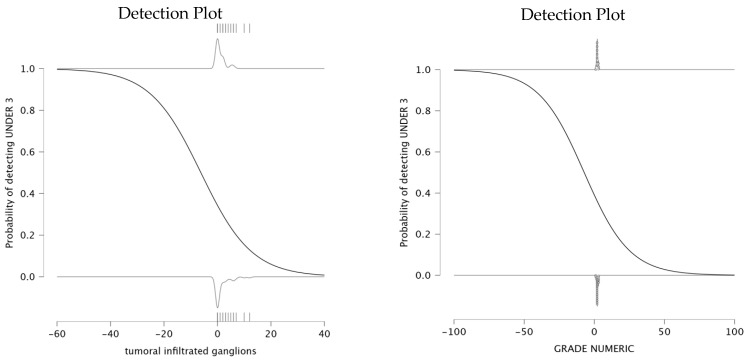
Probability of NLR over infiltrated lymph nodes and the probability of NLR to predict tumor differentiation.

The analysis explored the relationship between infiltrated lymph nodes and the probability of detecting an NLR under 3. The parameter estimates indicate a negative relationship between the number of infiltrated ganglions and the NLR value being under 3. The parameter estimates for this model show that the intercept is −0.649, and the coefficient for infiltrated lymph nodes is −0.105, suggesting that an increase in the number of infiltrated lymph nodes is associated with a lower probability of NLR remaining under 3. The detection plot (left panel of Figure 4) clearly illustrates this trend. This result suggests that as lymph node infiltration becomes more pronounced, systemic inflammation is likely to increase, which is reflected in elevated NLR values. Therefore, a higher number of infiltrated lymph nodes correlates with a higher value for NLR.

The analysis also evaluated the relationship between tumor differentiation and the value of NLR. The parameter estimates an intercept of −0.445 and a negative coefficient for grade (β = −0.062). The detection plot (right panel of Figure 4) shows a sharp decrease in the probability of detecting NLR < 3 as tumor grade increases. This suggests that patients with well-differentiated tumors are more likely to maintain a lower systemic inflammatory response, while higher tumor grades are associated with increased systemic inflammation, reflected in elevated NLR levels. This pattern supports the role of NLR as a potential biomarker for tumor differentiation and progression, with higher NLR levels being a possible indicator of poor prognosis.

**Figure 5:**
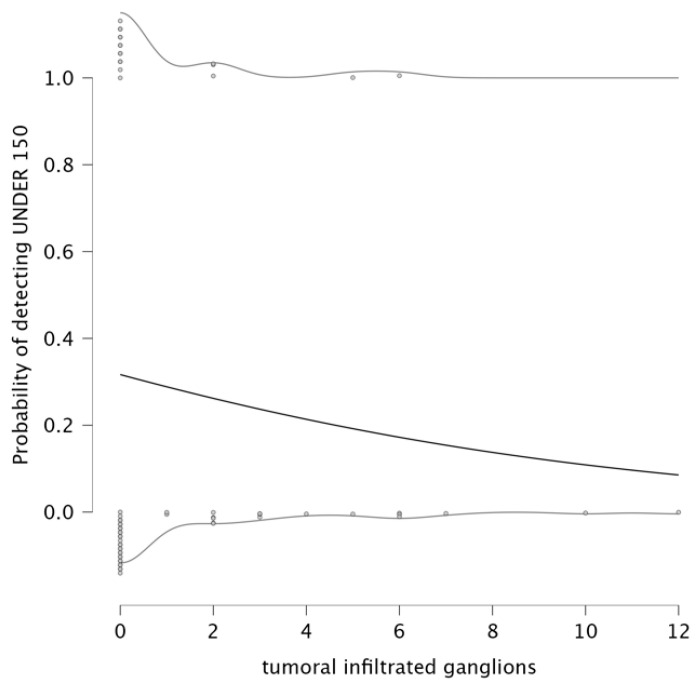
Detection plot analysis for PLR and number of infiltrated lymph nodes.

The analysis continued for PLR by setting the cut-off at 150, as previous studies mention [9,13,14]. A logistic regression model was used to determine the relationship between PLR and infiltrated lymph nodes. The results provided an intercept of −0.769 and a negative coefficient (β = −0.133). The data are presented as a detection plot in Figure 5. The upper portion of the plot shows that when positive lymph nodes are low (close to 0), the probability of detecting PLR < 150 remains high. This suggests that patients with minimal lymph node invasion tend to maintain lower PLR values, reflecting a lower systemic inflammatory response. As the number of infiltrated ganglions increases, the probability of detecting PLR under 150 steadily decreases. These findings suggest that PLR may be used as an inflammatory marker to assess tumor aggressiveness and lymphatic involvement.

**Figure 6:**
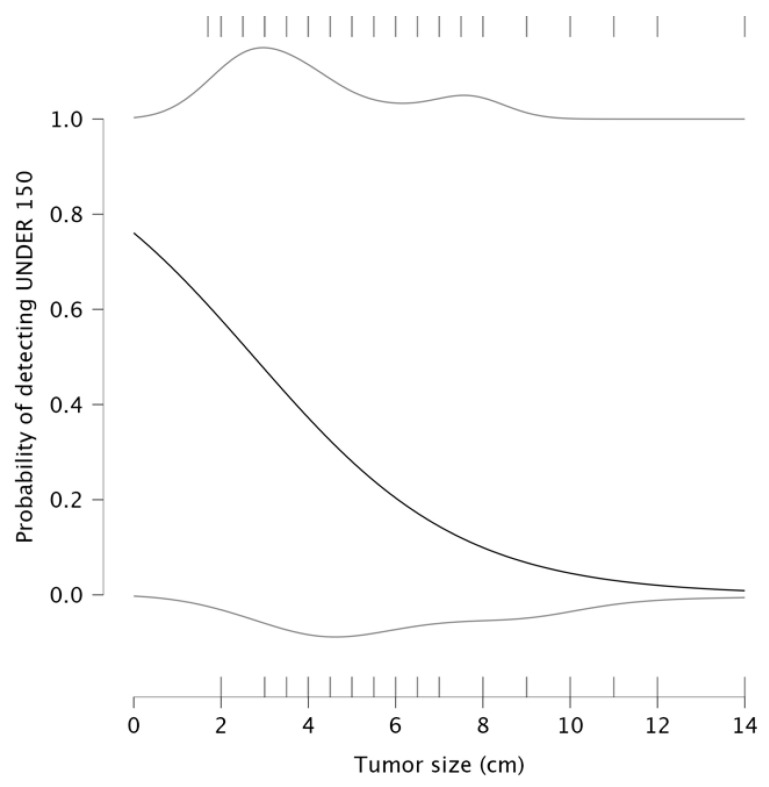
Detection plot; effect of tumor size on probability of PLR.

Another logistic regression was performed to assess the relationship between tumor size and the probability of having PLR below 150. The result suggests an inverse relationship between them, with an intercept value of 1.156 and a negative coefficient β = −0.420, meaning that when tumor size is smaller, the probability of having PLR < 150 remains relatively high, starting at approximately 0.8, as seen in the detection plot in Figure 6. The upper portion of the plot shows that for smaller tumors (under 2–3 cm), the probability of detecting PLR < 150 is relatively high (0.8–1.0). A gradual decline in probability occurs with an increase in tumor size beyond 4–5 cm. At tumor sizes exceeding 10 cm, the probability of detecting PLR under 150 approaches zero, meaning that patients with large tumors exhibit elevated PLR values. In this context, larger tumor sizes are associated with an immune response characterized by elevated PLR, implying that larger tumors might create an inflammatory environment conducive to tumor progression and metastasis.

**Figure 7:**
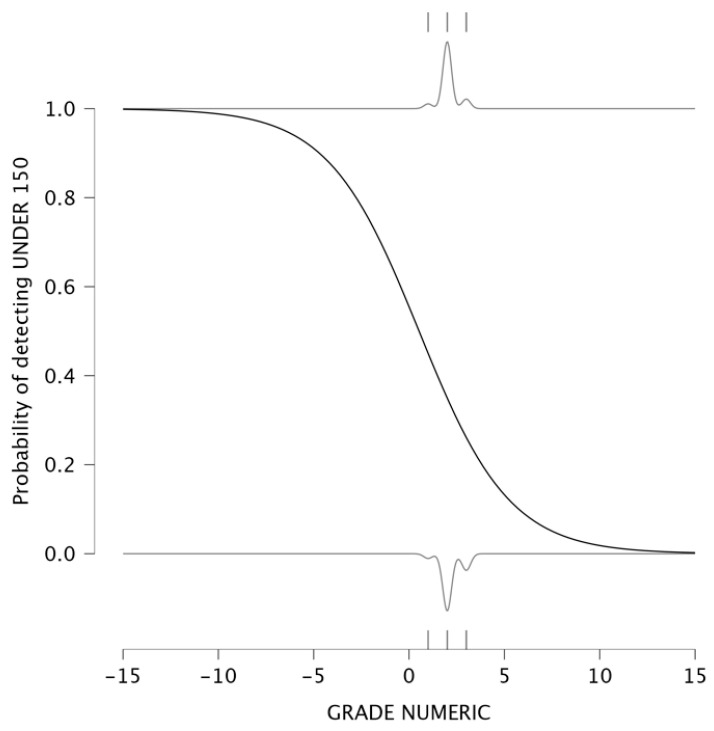
Detection plot. Probability of low PLR in relation to tumoral grading.

The investigation went further by evaluating the relationship between tumoral differentiation and the probability of having a PLR below 150. Tumor grading ranges from well-differentiated (lower grades) to poorly differentiated (higher grades), reflecting increasing tumor aggressiveness. A logistic regression model was used to analyze this relationship, resulting in an intercept of 0.221 and a negative coefficient β = −0.420. The detection plot is presented in Figure 7. The analysis demonstrated a strong inverse relationship between tumor grade and the probability of detecting PLR < 150. In low-grade tumors (well-differentiated), the probability of detecting PLR < 150 is close to 1, indicating that tumors with lower aggressiveness have weaker systemic inflammatory responses. For poorly differentiated tumors (G3 and G4), the probability of detecting PLR under 150 is nearly 0, meaning that patients with aggressive tumors exhibit PLR values above 150.

Given the borderline KMO and low NLR MSA, the component structure should be viewed as exploratory, not confirmatory.

## 4. Discussion

This study provides a direct comparison between the prognostic value of PLR and NLR in assessing tumor aggressiveness in colon cancer.

The findings of this study highlight a significant association between PLR and key tumor characteristics, including tumor size and staging, suggesting its potential utility as a prognostic biomarker. Conversely, NLR exhibited limited predictive value in this study, demonstrating no significant correlation with tumor size or other measures of disease severity. This study does not propose PLR or NLR as diagnostic markers for colon cancer. The findings of this study suggest that these inflammatory markers may serve as prognostic indicators for tumor aggressiveness and staging.

Research has shown that platelets release P-selectin adhesion factor, facilitating the adhesion of inflammatory cells to endothelial cells, which adversely affects tumor growth, metastasis, and prognosis [15,16,17]. Furthermore, platelets can release factors that stimulate tumor growth, driving tumor expansion [18]. In conclusion, PLR levels increase as cancer progresses [17]. Therefore, PLR’s stronger association with tumor size and staging may stem from the pro-tumorigenic role of platelets (release of P-selectin, platelet-derived growth factors).

PLR was significantly correlated with tumor size (r = 0.428, *p* < 0.001) and staging (r = 0.314, *p* = 0.012), reinforcing its role as an inflammatory biomarker. These findings align with previous research, which has suggested that increased PLR may indicate an enhanced inflammatory state that facilitates tumor progression and metastasis [19]. This study expands upon these findings, demonstrating clear relationship between PLR and tumor stages and suggesting that PLR may be used as an aid in preoperative risk stratification.

Furthermore, logistic regression analysis showed an inverse relationship between PLR and tumor differentiation, with low-grade tumors being more likely to exhibit elevated PLR values. This suggests that aggressive tumors provoke a stronger systemic inflammatory response.

In contrast, NLR did not demonstrate a significant correlation with tumor size or staging. Previous studies suggest that NLR may serve as a reliable marker for prognosis in colon cancer, but our findings indicate that its predictive value for tumor aggressiveness may be limited. However, PCA analysis revealed that NLR was primarily associated with systemic inflammation rather than direct tumor-related factors. Importantly, in this study, there were no patients with known active infections, autoimmune conditions, or hematologic disorders. The study did not control for all potential confounding factors that may influence NLR, such as undiagnosed chronic inflammation, metabolic syndrome, or other immune-modulating conditions. Future studies should further explore its role in stratified patient populations or in combination with molecular biomarkers.

The logistic regression models investigating PLR and NLR in relation to perineural and lymphovascular invasion did not yield statistically significant associations. This suggests that these markers are not able to directly predict more specific histopathological features of tumor invasion. These findings emphasize the complexity of tumor biology and the need for complementary biomarkers to refine risk assessment and prognosis.

The detection plots illustrated valuable insights into the prognostic significance of NLR and PLR. The probability of detecting NLR under 3 decreased significantly as tumor size increased. Similarly, PLR values exceeding 150 were consistently associated with larger tumors. These findings suggest that larger tumors induce a stronger systemic inflammatory response. The association between PLR and lymph node infiltration further supports its role in assessing tumor dissemination, as a higher number of infiltrated nodes corresponded to elevated PLR levels. Regarding tumor differentiation, poorly differentiated tumors (G3/G4) were more likely to exhibit increased NLR and PLR values, reflecting again a more aggressive inflammatory response. These findings suggest that PLR, rather than NLR, serves as a more reliable indicator of tumor progression and systemic inflammation. The detection plots underscore the potential of PLR as a non-invasive biomarker for tumor aggressiveness, particularly in preoperative risk assessment. A meta-analysis published in 2016 showed that seven studies reported significant correlation between PLR and tumor differentiation [20].

Given the relatively small sample size, PCA findings should be considered exploratory. Observed correlations were moderate and do not imply causation; they indicate potential relationships warranting prospective testing rather than clinical rule-in or rule-out behavior. A study published in 2023 found similar results, suggesting that an elevated PLR serves as an independent prognostic factor and is superior to NLR in predicting clinical outcomes in patients with colon cancer [20].

This study found a significant correlation between NLR and PLR (*p* < 0.001), indicating that these inflammatory markers are interrelated and likely reflect similar immune responses in colon cancer patients. This association suggests that both markers respond to tumor-driven inflammation, but their clinical utility differs. While PLR showed a clearer correlation with tumor size, staging, and aggressiveness, NLR appeared to be more reflective of general systemic inflammation rather than a direct predictor of tumor characteristics. This relationship highlights the importance of considering multiple inflammatory markers when evaluating tumor progression and underscores PLR’s superior prognostic potential in assessing disease severity.

The findings of this study have several important clinical implications for the management of colon cancer patients. These inflammatory markers may serve as valuable prognostic tools, particularly in the areas of preoperative risk stratification and treatment planning. It is important to emphasize that this study does not propose PLR or NLR as a diagnostic marker for colon cancer.

In clinical practice, routine preoperative blood tests already include platelet and lymphocyte counts, making PLR an easily accessible and cost-effective marker. Higher PLR values may indicate a more aggressive tumor, which could influence surgical planning by considering more extensive lymph node dissection and neoadjuvant therapy consideration. Surgeons could use PLR values to assess the risk of postoperative complications, especially in borderline resectable tumors.

Serial monitoring of PLR values postoperatively could provide additional insights into tumor recurrence and residual disease, helping identify patients at higher risk for metastatic progression. PLR could be incorporated in risk models for disease recurrence.

The limitations of this study must be acknowledged. The retrospective nature and the small cohort limit its applicability. The study included only 64 patients, so the small cohort reduces the generalizability of the findings. The small cohort limits statistical power, precision (wide CIs), and generalizability. Our models may be susceptible to overfitting, and the study was not powered for survival analyses or for detecting modest associations after multivariable adjustment. Large multicenter studies are needed to validate the results in diverse populations. The lack of survival analysis limits the ability to determine whether PLR or NLR has prognostic value for long-term survival. The survival analysis would be statistically underpowered on this small sample size. Also, while inflammatory markers provide valuable non-invasive insights, the lack of molecular specificity must be addressed to ensure accurate interpretations and to avoid the simplification of the complex tumor microenvironment. Future studies could integrate biomolecular analyses to better understand the systemic inflammation’s interaction with the tumor biology. The evaluation of PLR/NLR changes before and after surgery could provide further insights into tumor biology and surgical outcomes. A multicenter validation is needed before applying the results to broader clinical practice. Another limitation of this study is the lack of molecular profiling (e.g., RAS, BRAF and MIS status), which have a critical role in colon cancer prognosis. Although patients included had no documented infections, autoimmune diseases, or hematologic disorders, the study may still be subject to residual confounding from subclinical inflammatory conditions or metabolic syndromes, medications (steroids, NSAIDs), or smoking, all of which may alter leukocyte/platelet counts independent of tumor biology. Additionally, the PCA was performed under borderline sampling adequacy (KMO = 0.518) with low MSA for NLR and is presented strictly as an exploratory visualization rather than evidence of independent latent constructs.

Beyond PLR/NLR, other systemic factors may carry prognostic information. Essential elements (e.g., selenium, zinc, copper) have been linked to survival across multiple cancers, suggesting a broader host-metabolic context to outcomes [21]. Mechanistically, copper metabolism and the cuproptosis pathway provide a plausible link between trace-element status and tumor biology [22] Composite indices such as the systemic immune-inflammation index (SII) and systemic inflammation response index (SIRI) also show prognostic relevance in CRC and warrant evaluation alongside PLR in prospective designs [23].

The small sample size limits the generalizability of the findings and reduces statistical power in subgroup analyses. However, systemic inflammatory markers provide an independent perspective on tumor–host interactions and have potential value in complementing existing molecular prognosis models.

Future studies should focus on validating these findings in larger sample sizes and exploring the utility of NLR and PLR with other emerging markers for a more comprehensive predictive model. Also, the immunological basis must be investigated for these observations. Investigating the immunological mechanisms that drive the relationship between PLR and tumor aggressiveness will be essential for integrating these markers into routine clinical practice. Future research should explore the role of platelet-derived factors, such as P-selectin and platelet-derived growth factor, in tumor progression. Next-step studies should also integrate molecular profiling (RAS, BRAF, MSI status) and additional inflammatory indices (e.g., CRP, albumin, modified Glasgow Prognostic Score, systemic immune-inflammation index), enabling evaluation of PLR’s incremental value within modern prognostic frameworks. By expanding the understanding of the inflammatory response in colon cancer, there is an opportunity to improve treatment strategies and to enhance patient outcomes. The potential for PLR to guide immunotherapy decisions should also be investigated. Also, future research should explore integrating PLR into multimodal prognostic models among TNM staging and molecular markers to enhance risk stratification. A multicenter, prospective cohort with standardized blood sampling, adjudicated complications, and time-to-event outcomes (recurrence, disease-specific, and overall survival) is needed to evaluate whether PLR adds incremental prognostic value beyond stage, grade, lymphovascular/perineural invasion, and comorbidity burden.

While PLR may reflect elements of tumor burden, the observed effects are moderate and derived from a retrospective cohort. Accordingly, PLR should be considered a putative preoperative signal rather than a decision-making biomarker. Any clinical stratification based on PLR awaits external validation and demonstration of incremental value over established clinicopathologic predictors.

Because optimal thresholds vary across populations and laboratory platforms, we discourage dichotomization for clinical decision-making until externally validated cut-points are established in prospective datasets.

To implement PLR in routine clinical practice, cut-off values should be established through larger multicenter trials. PLR could be incorporated into risk stratification guidelines among the traditional staging parameters to enhance treatment strategies.

Observed associations for PLR were moderate and hypothesis-generating; they do not justify changes in surgical strategy or perioperative management without external validation and demonstration of incremental value over established clinicopathologic predictors.

## 5. Conclusions

This study provides preliminary evidence that the preoperative platelet-to-lymphocyte ratio (PLR) is a more reliable marker than the neutrophil-to-lymphocyte ratio (NLR) in assessing tumor aggressiveness in colon cancer patients. PLR showed consistent associations with tumor size, staging, and histologic grade, supporting its potential utility in preoperative risk stratification.

Unlike NLR, which may be more reflective of systemic inflammatory status in general, PLR appears to better capture tumor-related inflammation, possibly due to the pro-tumorigenic role of platelets in the tumor microenvironment. These findings emphasize the potential of PLR as a practical, non-invasive biomarker to assist surgical and oncological decision-making, particularly in healthcare systems with limited access to advanced molecular profiling.

Further prospective studies with larger, multicenter cohorts are warranted to validate these findings and to establish standardized cut-off values for clinical implementation.

These results should be interpreted as preliminary; no causal inference is implied, and no clinical use is advocated pending prospective validation.

## Figures and Tables

**Table 1:** Sociodemographic, clinical, surgical, and pathological characteristics of colon cancer patients.

	Frequency	Percent
Urban	41	64.063
Rural	23	35.938
Sex		
Male	42	65.625
Female	22	34.375
Tumor Location		
Sigmoid	29	45.313
Ascending colon	11	17.188
Descending colon	4	6.250
Transverse colon	11	17.188
Cecum	9	14.063
Surgery		
Right colectomy	25	39.063
Segmental resection	32	50
Left colectomy	7	10.93
Restoration of Intestinal Continuity		
Anastomosis	52	81.250
Stoma	12	18.750
Surgical Complications		
Anastomotic fistula	3	4.688
Right colectomy	1	1.562
Segmental resection	1	1.562
Left colectomy	1	1.562
Evisceration	2	3.125
Without complications	59	92.188
Anastomosis Type		
End to end	41	78.846
Side to side	8	15.384
End to side	3	5.769
Death during hospital stay	4	6.250
Metastasis		
Liver	8	12.500
Pulmonary	5	7.81%
Peritoneal	2	3.125
Local invasion	11	17.188
Staging		
I	12	18.750
II	23	35.938
III	18	28.125
IV	11	17.188
Tumoral Infiltraded Ganglions		
Not infiltrated	41	64.063
Infiltrated	23	35.937
Perineural Invasion		
Yes	20	31.250
No	44	68.750
Lymphovascular Invasion		
Yes	26	40.625
No	38	59.375
Grading		
Without (Mucinous)	14	21.875
G1	3	4.688
G2	38	59.375
G3	9	14.063
Histopathological Type		
NOS (not otherwise specified)	49	76.563
Mucinous	14	21.875
Multifocal	1	1.563

**Table 2:** Descriptive statistics of key clinical variables.

	Age (Years)	Length of Stay (Days)	Tumor Size (cm)	Infiltrated Lymph Nodes (Number)
Mean	70.469	11.953	5.612	1.46
Standard deviation	10.408	6.090	2.700	2.600
IQR	12.500	6.00	3.625	2.000
Minimum	38.000	3.00	1.700	0.000
Maximum	90.000	44.000	14.000	12.000

**Table 3:** Descriptive statistics of preoperative blood markers in colorectal cancer patients.

	Mono	PLT	HGB	WBC	LYM	NEU	NLR	PLR
Mean	0.709	313.653	10.739	9.895	1.551	7.413	6.015	237.204
Standard deviation	0.406	130.928	3.243	5.150	0.670	4.921	6.430	135.990
IQR	0.454	147.250	4.995	4.558	0.870	4.185	3.762	171.762
Minimum	0.210	101.000	3.810	3.660	0.543	2.020	1.600	38.260
Maximum	2.310	774.000	15.770	34.370	3.850	31.420	36.930	670.150

**Table 4:** Pearson’s correlation between NLR, PLR, and tumor characteristics.

Variable		NLR	PLR
PLR	Pearson’s r *p*-value	0.536 <0.001	-
Length of stay	Pearson’s r *p*-value	−0.042 0.740	−0.049 0.701
Complications	Pearson’s r *p*-value	−0.124 0.330	−0.111 0.384
Death during hospital stay	Pearson’s r *p*-value	−0.016 0.898	−0.068 0.594
Tumor grading	Pearson’s r *p*-value	−0.049 0.737	−0.025 0.865
Tumor size	Pearson’s r *p*-value	0.221 0.079	0.428 <0.001
Infiltrated lymph nodes	Pearson’s r *p*-value	0.079 0.537	0.071 0.577
Staging	Pearson’s r *p*-value	0.154 0.223	0.314 0.012
Metastasis	Pearson’s r *p*-value	−0.038 0.764	0.111 0.382

**Table 5:** Analysis of covariance (ANCOVA) results. Length of stay.

Cases	Sum of Squares	F	*p*	ω^2^
Death during hospital stay * Surgical complications	387.072	25.888	<0.001	0.131
NLR	0.911	0.061	0.806	0
PLR	0.036	0.002	0.961	0
Death during hospital stay	261.817	17.511	<0.001	0.087
Surgical complications	1303.611	87.189	<0.001	0.454

* covariates in ANCOVA model.

**Table 6:** Assumption checks.

Test for Equality of Variances (Levene’s)			
F	df1	df2	*p*
1.139	3.000	60.000	0.341

**Table 7:** PCA analysis.

Component Loadings			
	RC1	RC2	Uniqueness
Grade (*n*)	0.866		0.305
Tumor size (cm)	0.660		0.567
Infiltrated lymph nodes	0.495	0.469	0.409
NLR cut-off		0.960	0.143

*Note*. Applied rotation method is Promax.

## Data Availability

The datasets generated and analyzed in this study are not publicly available due to confidentiality restrictions but may be available from the corresponding author on reasonable request and with appropriate institutional approvals.
